# A Comparison of the Monetized Impact of IQ Decrements from Mercury Emissions

**DOI:** 10.1289/ehp.9797

**Published:** 2007-02-26

**Authors:** Charles Griffiths, Al McGartland, Maggie Miller

**Affiliations:** U.S. Environmental Protection Agency, Washington, DC, USA

**Keywords:** benefits, CAMR, dose response, fish consumption, IQ, mercury, methylmercury, U.S. EPA

## Abstract

**Objective:**

The U.S. Environmental Protection Agency (EPA) reports that the upper bound of benefits from removing mercury emissions by U.S. power plants after implementing its Clean Air Interstate Rule (CAIR) is $210 million per year. In contrast, Trasande et al. [Environ Health Perspect 113:590–596 (2005)] estimated that American power plants impose an economic cost of $1.3 billion due to mercury emissions. It is impossible to directly compare these two estimates for a number of reasons, but we are able to compare the assumptions used and how they affect the results.

**Data Sources and Data Extraction:**

We use Trasande’s linear model with a cord/maternal blood ratio of 1.7 and calculate health effects to children whose mothers had blood mercury levels ≥ 4.84 μg/L.

**Data Synthesis:**

We introduce the assumptions that the U.S. EPA used in its Clean Air Mercury Rule (CAMR) analysis and discuss the implications. Using this approach, it is possible to illustrate why the U.S. EPA assumptions produce a lower estimate.

**Conclusions:**

The introduction of all the U.S. EPA assumptions, except for those related to discounting, decreases the estimated monetized impact of global anthropogenic mercury emissions in the Trasande model by 81%. These assumptions also decrease the estimated impact of U.S. sources (including power plants) by almost 97%. When discounting is included, the U.S. EPA assumptions decrease Trasande’s monetized estimate of global impacts by 88% and the impact of U.S. power plants by 98%.

## Background

On 15 March 2005, the U.S. Environmental Protection Agency (EPA) promulgated the Clean Air Mercury Rule (CAMR), which is the first federal rule—and the first rule in the world—to permanently cap and reduce mercury emissions from coal-fired power plants. During the final stages of promulgating this rule, Trasande et al. (2005) published an article titled “Public Health and Economic Consequences of Methyl Mercury Toxicity to the Developing Brain,” which raised some issues regarding how to measure certain benefits that society will receive from reducing mercury emissions. Trasande et al. (also, hereafter, Trasande) analyzed the economic costs of methylmercury toxicity from anthropogenic mercury emissions, measured as a decrease in IQ. They reported that the monetized cost of global anthropogenic mercury emissions amount to $8.7 billion [range, $4.9–$13.9 billion (in 2000 dollars)] per year, of which they claimed $1.3 billion (range $100 million–$6.5 billion) can be attributed to American power plants. The authors have subsequently revised these ranges to $0.7–$13.9 billion for global anthropogenic emissions and $51 million–$2.0 billion for U.S. power plants (Trasande et al. 2006b). In contrast, the U.S. EPA has reported that the upper bound of benefits resulting from removal of mercury emitted by U.S. power plants after implementing its Clean Air Interstate Rule (CAIR) is $210 million per year (U.S. EPA 2005c, 2006).

## Objective

Although the U.S. EPA value does fall within Trasande’s range, the difference between Trasande’s primary estimate and the U.S. EPA’s estimate is striking. This contrast raises the question of whether or not the two values can be compared. Stated briefly, it is impossible to directly compare these two estimates because both the approach used and the amount of mercury assumed are fundamentally different in the two analyses. However, because the economic end point analyzed (i.e., reduced IQ) is the same, we can compare the assumptions used and how they influence the results. Using the Trasande approach, we illustrate why the assumptions employed by the U.S. EPA produce a lower estimate.

## Approaches

### Trasande approach

For their analysis, Trasande et al. (2005) focused on decrements in intelligence quotient (IQ) associated with prenatal mercury exposure. They used an environmentally attributable fraction model to estimate the damages done by exposure to anthropogenic sources of mercury. This model is specified as:


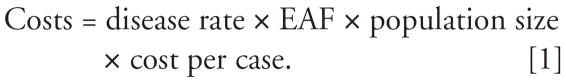


Trasande derived the disease rate using one of two dose–response estimates (a logarithmic and a linear estimate) from an epidemiologic study of prenatal mercury exposure in the Faroe Islands (Budtz-Jorgensen et al. 2004a; Grandjean et al. 1997). The exposed population is based on data from the National Health and Nutrition Examination Survey (NHANES) (Mahaffey et al. 2004), and the cost per case is derived from a reduction in lifetime earnings (based on Max et al. 2004) from reduced IQ (Salkever 1995). The additional EAF is the “environmentally attributable fraction” (Smith et al. 1999), a factor to proportionally allocate costs to a particular environmental cause. Because Trasande et al. were concerned with estimating the costs associated with anthropogenic sources of mercury, the EAF is simply the portion of mercury that can be attributed to human activities and was set at 70% of the total global mercury pool (Mason and Sheu 2002; U.S. EPA 1997b).

Using their chosen parameters, Trasande et al. (2005) reported that the cost to the United States of global anthropogenic mercury emissions ranges from $2.2 to $43.8 billion (2000 dollars), with their preferred estimate of $8.7 billion based on the logarithmic model. They further reported an estimate of the cost of American anthropogenic emissions by multiplying their global value times a weighted average of U.S. mercury content in all fish. Trasande et al. derived this weighted average by estimating the contribution of U.S. emissions to both domestically caught and imported fish. The authors reported that the cost of U.S. anthropogenic emissions ranges from $400 million to $15.8 billion. Finally, the authors reported an estimate of the cost of U.S. power plant emissions, ranging from $100 million to $6.5 billion, by multiplying their U.S. cost figures times the percent of American emissions attributable to American power plants. In a subsequent analysis, the authors revised the ranges downward, but their preferred estimates remained the same as in their original analysis (Trasande et al. 2006b).

### U.S. EPA approach

For CAMR, promulgated in March 2005, the U.S. EPA used a spatially explicit model of air quality to determine the location of mercury deposition from U.S. power plants. On the basis of models of fishing behavior, the U.S. EPA evaluated the benefits from what it considered to be the most important environmental pathway for mercury exposure: prenatal exposure from the consumption of recreationally caught freshwater fish. In March 2005, the U.S. EPA reported monetized benefits from implementing CAMR, measured as decreases in IQ, of $0.8–$3.0 million per year (U.S. EPA 2005c). For several reasons, including the fact that the U.S. EPA estimated the impact from only a single pathway for methylmercury toxicity, the U.S. EPA was petitioned to reconsider CAMR. In the technical support document for this reconsideration, the U.S. EPA estimated an upper bound for the potential benefits that could possibly be obtained from CAMR, considering all exposure pathways. The U.S. EPA accomplished this by estimating the benefits from removing all remaining mercury emissions from U.S. power plants after the implementation of CAIR. CAIR is a U.S. EPA rule, promulgated before CAMR, which also reduced mercury emissions as a co-benefit. The U.S. EPA’s upper-bound estimate, published in its response to comments on the reconsideration notice, was $210 million per year (U.S. EPA 2006).

### No direct comparison

Because Trasande et al. (2005) evaluated the economic costs of IQ decrements due to mercury exposure, and the U.S. EPA estimated the benefits of reducing IQ decrements due to CAMR, it is tempting to compare these two results. The reasoning is that if Trasande et al. correctly estimated the cost that U.S. power plants place on society, then this is an estimate of the benefits of CAMR. In fact, it was the very large values initially reported by Trasande et al. and the small values reported by the U.S. EPA that made some question the underlying assumptions. A direct comparison between these analyses, however, is not appropriate for a number of reasons.

First, Trasande et al. were evaluating the benefits of eliminating all anthropogenic mercury and then parsing out how much is attributable to U.S. power plants, whereas CAMR only reduces 70% of mercury emissions from U.S. power plants. Under CAMR, coal-fired power plants will be required to reduce emissions from their current level of 48 tons/year to a maximum of 15 tons of mercury per year beginning in 2018, but mercury emissions are not totally eliminated. Furthermore, on 10 March 2005, 5 days before promulgating CAMR, the U.S. EPA issued CAIR, which was designed to permanently cap emissions of sulfur dioxide and nitrogen oxides from American power plants. One of the additional benefits from CAIR, its so-called “co-benefits,” is that the technology used to reduce SO_2_ and NO_x_ will also reduce mercury emissions. Therefore, a portion of the benefits from reduced mercury emissions over the next couple of years will be attributed to the implementation of CAIR. The correct measure of benefits from CAMR reflects the difference between the state of affairs after the implementation of CAIR and the state of affairs with 15 tons of mercury emissions. This is quite different from the elimination of all current mercury emissions.

Second, even if we were to take the proportion of mercury reduced under CAMR as a proportion of the total reported by Trasande et al., the environmentally attributable fraction model is a less complex approach than the U.S. EPA’s economic analysis for CAMR. The U.S. EPA modeled the location of mercury deposition using a spatially explicit air quality model to assess the magnitude of fish contamination and a behavioral model to assess population consumption patterns of these fish (U.S. EPA 2005b). However, this more sophisticated approach was applied only to the consumption of recreationally caught freshwater fish. One advantage of the fractional approach used by Trasande et al. is that it can be applied to all exposure pathways, but it does so by assuming that fish contamination levels and consumption patterns are uniform across the United States. In short, it is impossible to directly compare these two analyses because they use two fundamentally different approaches (U.S. EPA uses a deposition model and Trasande uses a fractional model), and they do not estimate impacts for the same populations (U.S. EPA’s analysis addresses only a subset of the total because it estimates the mercury reduction solely attributable to CAMR).

Third, the Trasande approach does not account for either the response time in implementing mercury reductions or the response time of the environment to these reductions. As mentioned above, because of prior mercury reductions from CAIR, benefits from CAMR do not begin until the implementation of the 15-ton cap in 2018. Additionally, the ecosystem takes time before reductions in the air deposition of mercury are translated into decreases in methylmercury in fish tissue. This environmental response time has been estimated to be on the order of decades before the benefits of mercury reductions are fully realized. In short, the Trasande estimates cannot be construed as a measure of the benefits from regulatory actions to reduce mercury. At best, they would be an estimate of the impact of the instantaneous elimination of all anthropogenic mercury from the environment.

Although the two analyses cannot be compared directly, there may be some utility from understanding the assumptions used by both Trasande et al. and the U.S. EPA. A careful, well-reasoned assessment of the current costs imposed by all anthropogenic mercury exposure in the United States could serve as a possible starting point for a discussion of the benefits of reducing mercury. In what follows, we discuss the difference in assumptions used by Trasande et al. and the U.S. EPA. We then use one of the models presented by Trasande et al. and introduce the assumptions that the U.S. EPA incorporated in its CAMR analysis while discussing the implication of introducing these assumptions.

## Assumptions

### Model choice

The model we use for our comparison is Trasande’s linear model, with a cord:maternal blood ratio of 1.7 and calculated health effects to children whose mothers have a blood mercury level of ≥ 4.84 μg/L. This model is presented by Trasande et al. (2005) as a sensitivity analysis rather than the primary analysis that uses a logarithmic model of the dose–response relationship. We chose to use the sensitivity analysis model for the comparison for two reasons. First, a logarithmic model assumes that there is a supralinear relationship between mercury exposure and IQ decrements. However, the National Research Council (NRC) has recommended the use of a linear dose–response relationship and explicitly cautioned against using a log-transformed (i.e., supralinear) dose–response function to portray the mercury exposure–IQ relationship. Specifically, the NRC (2000) said

After extensive discussion, the committee concluded that the most reliable and defensible results for the purpose of risk assessment are those based on the K-power model. … Both the square-root and the log models take on a supralinear shape at low doses, that is, they postulate a steeper slope at low doses. … From a toxicological perspective, the K-power model has greater biological plausibility, because it allows for the dose response to take on a sublinear form, if appropriate. … The K-power model is typically fit under the constraint that K ≥ 1, so that supralinear models are ruled out.

Given this recommendation, the U.S. EPA used a linear dose–response function in its analysis. Therefore, Trasande’s linear model is the only one that is appropriate for a direct comparison. Second, the assumptions that *a*) there is a cord:maternal blood ratio of 1.7 and *b*) negative health effects occur only when the mother’s blood mercury level is ≥ 4.84 μg/L produce the highest values of all of the linear models. Therefore, our discussion can revolve around the upper bound of the costs of anthropogenic mercury exposure, assuming a linear dose–response relationship.

### Dose–response slope for cord blood measurement

Trasande’s linear model originally used a dose–response relationship of 0.59–1.24 IQ point decrements for every 1-μg/L increase in cord blood mercury concentration, with an average decrement of 0.93 IQ points (Trasande et al. 2005). Trasande et al. (2006b) have since changed this value to a corrected dose–response value of 0.093. Therefore, we use the corrected value of −0.093 IQ points for each 1-ppb increase of mercury in cord blood (referred to as “Trasande (corrected)” in the data tables below) as the mean estimate of the linear dose–response slope for use in recalculating Trasande’s estimates.

For its approach, the U.S. EPA used a statistical analysis to integrate data from the three major studies investigating the potential neurotoxicity of low-level, chronic mercury exposure: the New Zealand study (Crump et al. 1998; Kjellstrom et al. 1989), the Seychelles Child Development Study (Davidson et al. 1998; Myers et al. 2003), and the Faroe Isands study (Budtz-Jorgensen et al. 2004a; Grandjean et al. 1997). The integrated statistical analysis (Ryan 2005) produced a dose–response relationship with a central estimate of −0.16 IQ points per part per million of mercury in hair (U.S. EPA 2006). Using a value of 200 as the ratio of mercury in hair to mercury in cord blood for the Faroe Islands cohort (Budtz-Jorgensen et al. 2004b), the U.S. EPA’s dose–response value implies a relationship of −0.032 IQ points for each 1 ppb mercury in cord blood, substantially lower than the value used by Trasande et al.

We note that the corrected Trasande value of −0.093 IQ points for each 1 ppb of mercury in cord blood implies a 0.465 IQ decrement for each part per million of mercury in hair. Although this value is in the range of what has been found in some studies, it is on the high end. By comparison, Ryan’s (2005) evaluation of the Faroe Islands data, the same data set used by Trasande, indicates a linear dose–response relationship of −0.12 IQ points per part per million mercury in maternal hair.

### Lifetime earnings

For both Trasande and the U.S. EPA, a decrement in IQ was translated into a decrease in lifetime earnings. We note that lost earnings from IQ loss is not the conceptually correct metric for valuing benefits of reduced mercury exposure. Ideally, one should use a measure of willingness-to-pay to avoid neurobehavioral damage caused by mercury exposure. However, there is currently no acceptable estimate of willingness-to-pay in the economics literature, so both analyses rely on the change in earnings resulting from a change in IQ.

Trasande et al. used a value for lifetime earnings (in 2000 dollars) of $1,032,002 for males and $763,468 for females based on the work of Max et al. (2004). These values were calculated from the mean annual earnings for full-time, year-round workers in 5-year intervals (Arias 2002), adjusted upward by 1.6 for wage supplements (e.g., fringe benefits and employer contribution to insurance benefits) and supplemented with a small additional amount for the imputed value of household production. Trasande et al. multiplied this earnings figure by a labor force participation rate based on the percent of the population whose major activity in the preceding week was working at a job or business, as reported in the 2000 National Health Interview Survey. The authors then summed the earnings across age intervals, assuming a 3% discount rate and a 1% annual gain in productivity.

The U.S. EPA estimated the average present value of future earnings using the total average annual earnings for the population, also in 5-year intervals, broken out by sex and education level as reported in the 1992 Current Population Survey (U.S. Department of Commerce 1993). The U.S. EPA also summed the earnings across age intervals, assuming a 3% discount rate and a 1% annual gain in productivity. For both sexes combined, U.S. EPA reports total lifetime earnings of $366,021 in 1992 dollars (U.S. EPA 2000). Using a GDP deflator, this would imply a value of $472,465 in 2000 dollars.

There appear to be two major differences between the U.S. EPA’s value and the Trasande value. First, the U.S. EPA value does not appear to include wage supplements and household production values, an important consideration. Second, the U.S. EPA used total average earnings for the population rather than multiplying a participation rate by the earnings for full-time workers to arrive at average earnings.

Both the U.S. EPA and Trasande use the work of Salkever (1995) to estimate the effect that one IQ point decrement has on earnings. Because the Trasande earnings are sex specific, the authors use Salkever’s sex-specific results. For each IQ point decrement, males experience a 1.93% decrease in lifetime earnings and females experience a 3.23% decrease. The U.S. EPA used a participation-weighted average of 2.379% for the combined lifetime earnings figure. One other important distinction between the two analyses is that, following Salkever, U.S. EPA adjusted their dollar value per IQ point for the savings associated with reduced years in schooling, whereas Trasande et al. appear not to have made this adjustment. Although this adjustment may be correct, it is difficult to implement in replicating the Trasande analysis, so it is not included in future discussions.

### Percent of fish consumption affected by U.S. sources

In determining the amount of mercury in fish attributable to U.S. sources, Trasande et al. note that 42% of the supply of edible fish in the U.S. is imported (National Marine Fisheries Service 2003) and they estimate that 2% of the mercury content of imported fish is attributed to American anthropogenic sources. The remaining 58% of the U.S. fish supply is not imported. Using 1995 emission estimates from the U.S. EPA’s *Mercury Study Report to Congress* 1997b, Trasande et al. estimate that 87 tons of mercury were deposited on U.S. soil in 1995, 60% of which came from U.S. anthropogenic sources. They then attribute this 60% contribution from U.S. sources to the 58% of the domestically caught fish supply. Combining the assumptions for both domestic and imported sources, this implies that approximately 36% of mercury exposure from fish consumption is attributed to U.S. anthropogenic sources [i.e., (0.42 × 0.02) + (0.58 × 0.60) ≈ 0.36]. Using data from the 1999 National Emissions Inventories for Hazardous Air Pollutants (U.S. EPA 2003), Trasande et al. estimate that 41% of the U.S. anthropogenic sources comes from U.S. electric power plants, thereby implying that 15% (i.e., 0.41 × 0.36) of all mercury exposure from fish consumption is attributed to U.S. electric utilities.

There are a number of reasons to question some of these values. The estimate that 58% of the U.S. edible fish supply is domestically caught is based on landings data. However, marine species comprise approximately 96% of the market share of seafood, which includes freshwater and marine fin and shell fish (Carrington et al. 2004). Many of these marine species spend at least part of their life cycle in the open ocean, so their mercury content is likely influenced more by the global mercury pool than by domestic deposition. Another problem is in determining the location of where the fish are caught. For example, it is highly unlikely that 60% of mercury content in pollock, which has an 11% share of the seafood market, can be attributed to U.S. anthropogenic sources. Well over 95% of the pollock supply in the U.S. is Alaskan pollock from the Pacific Ocean, but U.S. power plants are located east of the Pacific and the prevailing winds in the United States are easterly. Ultimately this means that mercury from the emissions will be depositing east of the Pacific, away from Alaskan pollock populations. Similarly, over 90% of the cod supplied in the United States is Pacific cod.

As mentioned above, in evaluating the impact of mercury emissions, the U.S. EPA used a spatially explicit air quality model to simulate the location of mercury deposition. This makes a comparison between Trasande’s estimate of fish consumption affected by U.S. sources and the U.S. EPA’s estimate very difficult. At best, the Trasande model can be thought of as a special case of the spatially explicit U.S. EPA model, one where many or all of the spatially differentiated variables are assumed equal. However, to evaluate the impact of different assumptions, we can use the U.S. EPA’s approximation of the total mercury deposition in the United States as estimated for CAMR reconsideration. The U.S. EPA estimated that 144 tons of mercury was deposited in the continental United States in 2001, and that 121 (or 84%) came from sources outside of the United States and Canada (U.S. EPA 2005a). This means that, on average, 16% of the total mercury deposition in the United States comes from American and Canadian sources. Again, this value is an average value and does not reflect the percentage content of mercury in American freshwater fish that can be attributed to American sources. It does, however, provide an U.S. EPA assumption equivalent to that used by Trasande et al.

To estimate the portion of consumption affected by the domestic deposition of mercury, we use the U.S. EPA’s upper-bound estimate in the *Technical Support Document* for CAMR reconsideration (U.S. EPA 2005c). This analysis applied the consumption rates from the U.S. EPA’s *Exposure Factors Handbook* (U.S. EPA 1997a), which recommends using a mean consumption rate of 20.1 g fish/day for the general population. Of this 20.1 g fish/day, 70% (14.1 g) is associated with the consumption of marine fish and 30% (6 g) is associated with freshwater, estuarine, or aquaculture fish consumption. Following Trasande et al., we can assume that the 70% is affected by the global pool and 30% is affected by U.S. anthropogenic emissions.

### Ecosystem adjustment

The last large disparity in assumptions between the U.S. EPA and Trasande et al. is in the accounting for the ecosystem response time. As mentioned previously, the Trasande analysis can be characterized as an estimate of the economic costs associated with IQ decrements due to anthropogenic mercury exposure, whereas the U.S. EPA’s analysis estimates the benefits from reductions in mercury emissions. Also, although it is tempting to interpret the Trasande results as estimates of benefits, this would be wrong. A proper economic benefits analysis must account for the timing of the impacts, which in this case involves the response time the ecosystem needs to manifest mercury reductions in fish tissue.

Estimating this response time is difficult because different ecosystems exhibit dramatically different responses to changes in mercury loading, depending on their chemical and physical attributes. Among the five freshwater ecosystems investigated by U.S. EPA for CAMR, the time required for mercury to reach equilibrium after a decrease in mercury loading (measured as reaching 90% of its steady-state level) ranged from < 5 years to ≥ 30 years (U.S. EPA 2005b). The time required for ocean environments to reach steady state can range from approximately 30 years for the Atlantic Ocean to as much as 200 years for the Pacific Ocean (U.S. EPA 2005c). Naturally, benefits will build over time during the transition path from the current conditions to the new equilibrium, but they are not immediate. This transition path can be represented by choosing an average period over which to discount the benefits. For the *Technical Support Document* for the CAMR reconsideration (U.S. EPA 2005c), the U.S. EPA used an average 15-year response lag with a 3% discount rate.

## Model Comparison

Table 1 lists the results of Trasande’s base case linear model, using the corrected dose–response slope and a cord:maternal blood ratio of 1.7. Each segment of the child-bearing population, with a blood mercury concentration level of ≥ 4.84 μg/L, is assigned to one of four categories reflecting the current U.S. blood mercury level distributions. This segment of the population constitutes approximately 8% of the total child-bearing population. The change in concentration from a total elimination of mercury exposure is then calculated, assuming a no effect concentration of 3.41 μg/L. This means that children whose mothers had a blood mercury level ≤ 3.41 μg/L are assumed to have no negative health effects attributable to mercury.

Multiplying the change in concentration by the dose–response slope produces the estimated number of IQ points lost due to the given mercury exposure. Multiplying the estimated lifetime earnings by the percentage change in earnings per loss of IQ point gives the monetized cost of one IQ point for both boys and girls. Multiplying the number of IQ points lost by the cost of an IQ point and the number of children of each sex gives the overall monetized cost of mercury exposure. Multiplying this number by the EAF produces an estimate of the impact that global anthropogenic mercury exposure has on U.S. children. Summing the costs across the four analyzed segments of the population produces a base case estimate of approximately $3 billion from Trasande’s linear model.

Table 2 provides a comparison of the monetized impact of IQ decrements from anthropogenic mercury emissions under the assumptions used by Trasande et al. and by the U.S. EPA. The numbers have been rounded to avoid false precision. The first column of numbers lists the values originally reported by Trasande et al. (2005). The second column of numbers lists the Trasande values with the corrected dose–response coefficient of −0.093. In the second column, the undiscounted monetized impact of anthropogenic emissions is approximately $3 billion.

Following the logic of Trasande et al. (2005), the impact of U.S. anthropogenic emissions is found by multiplying this $3 billion by the weighted sum of fish consumption affected by U.S. sources. This weighted sum is equal to:


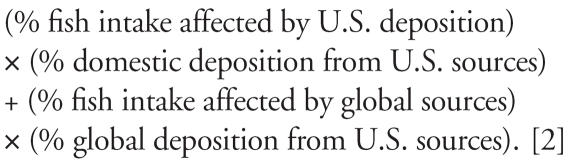


By Trasande’s calculation, U.S. sources have a monetized impact of approximately $1 billion. The impact specifically attributable to U.S. power plants is approximately $480 million. This number is found by multiplying the monetized impact of U.S. sources by the percent of U.S. emissions attributed to U.S. power plants (41%). The discounted effects numbers in column 1 and column 2 are repeats of the undiscounted values because Trasande et al. (2005) do not include discounting. These numbers are simply shown for comparison purposes with the U.S. EPA assumptions in column 3. The “Assumptions” section of the table lists the assumptions that were introduced to derive these values. The only change between the first and second column of numbers is the linear dose–response slope, which reduces the original values by an order of magnitude.

The final column of numbers shows what this model would produce if the U.S. EPA assumptions, listed in the lower section, were introduced. Using all of the U.S. EPA’s assumptions in this model, except the discount rate and ecosystem response time lag, produces an estimate of the undiscounted effects. The undiscounted monetized impact of all global anthropogenic emissions would be on the order of $580 million, or about 20% of that reported by Trasande et al. The undiscounted monetized impact of U.S. anthropogenic emissions is approximately $35 million, and the impact of U.S. power plants is around $15 million.

As discussed previously, these monetized impacts could be translated into benefit values only if the discount rate and time for ecosystem adjustment were included. Introducing the U.S. EPA’s assumptions of a 3% discount rate and an average 15-year ecosystem adjustment period produces the discounted effects reported in the last column. The discounted impact of all global anthropogenic emissions is estimated to be approximately $370 million. Of this, the discounted impact of U.S. anthropogenic emissions is approximately $25 million, with the impact of U.S. power plants estimated to be around $10 million.

As the results in Table 2 show, the impact of introducing all of the U.S. EPA’s assumptions, except for those related to discounting, would decrease the estimated monetized impact of anthropogenic emissions in the corrected Trasande model by 81%. It would also decrease the estimated impact of U.S. sources (including power plants) by almost 97%. Including discounting makes the difference even starker; the U.S. EPA’s assumptions decrease Trasande’s estimate of global impacts by 88% and U.S. power plant impacts by 98%.

These results are derived when all of the U.S. EPA’s assumptions are applied as a whole. Table 3 illustrates the impact of introducing the U.S. EPA’s assumptions individually. The “Global estimate” column lists the percentage decrease in Trasande’s estimated impact of global anthropogenic emissions from introducing a single U.S. EPA assumption. The “U.S. estimate” column lists the percentage decrease in Trasande’s monetized impact of U.S. anthropogenic emissions from introducing a single U.S. EPA assumption.

One of the larger percentage changes occurs as a result of using the U.S. EPA’s dose–response slope of −0.032 instead of the corrected Trasande estimate of −0.093. This change alone reduces the estimate of the undiscounted monetized impact of all global anthropogenic emissions by 66%. This change does not affect the estimate of the impact from U.S. anthropogenic emissions in any way other than through its reduction in global impacts.

The next model component evaluated in Table 3 is the choice of a lifetime earnings value. The lower value used by the U.S. EPA reduces Trasande’s global estimate by 46% and, as with the dose–response curve, does not have any additional effect on the estimate of the impact of U.S. sources. This impact comes almost exclusively from a difference in the reported lifetime earnings value. This earnings value is a base to which the loss associated with an IQ decrement is multiplied. Although Trasande used sex-specific weights for earnings loss and the U.S. EPA used a participation-weighted factor for the whole population, the impact of substituting the U.S. EPA assumption is small, decreasing Trasande’s results by approximately 4%.

Changing the percent of fish consumption directly affected by global and domestic deposition does not affect the total monetized impact of anthropogenic emissions from all global sources. However, changing these percents does affect the estimated monetized impact associated with U.S. anthropogenic emissions, including those from U.S. power plants. Estimating the percent of fish consumption affected by domestic deposition using consumption patterns, as is done by the U.S. EPA, rather than using landings data, reduces Trasande’s estimate of the impact of U.S. sources on fish consumption by 46%.

The U.S. EPA assumption that creates the largest contrast when compared to Trasande’s results is the percent of domestic deposition attributable to U.S. sources. Using its air quality model, the U.S. EPA estimated that U.S. sources are responsible for 16% of the mercury deposition in the continental United States, as opposed to the 60% assumed by Trasande. This change alone reduced Trasande’s estimate of the impact of American sources by 72%. As with the estimate of the percent of fish consumption affected by U.S. sources, noted above, this change does not affect the global estimate. The percent of emissions attributable to power plants was kept the same for this exercise, so it does not affect either the global or the U.S. results.

The final two assumptions pertain to the discount rate and the average ecosystem response time. Introducing the two U.S. EPA assumptions alone decreases the corrected Trasande results by 36%.

As described above, introducing all of the assumptions together decreases the undiscounted global impacts by 81% and the U.S. impacts by 97%, and decreases the discounted results by 88% and the U.S. impacts by 98%.

## Summary and Discussion

This analysis shows that the impact of introducing the U.S. EPA assumptions into the Trasande model produces dramatic changes in the monetized impact. In our view, the U.S. EPA assumptions are more appropriate than those of Trasande et al.

The first important decision concerns model choice. The base case model presented by Trasande et al. is one which assumes a logarithmic dose–response relationship between IQ decrements and mercury exposure. Although Budtz-Jorgensen et al. (2004a) did present both a logarithmic model and a linear model for the Faroe Islands results, the NRC (2000) explicitly argued against using a supra-linear (e.g., logarithmic) model for mercury exposure. Therefore, the linear model seems to be the more appropriate model for this analysis.

As can be inferred from Table 3, the choice of the dose–response curve slope is extremely important to the overall results. We believe that a statistical analysis incorporating the data from the three major studies investigating the potential neurotoxicity of low-level, chronic mercury exposure (New Zealand, the Seychelles, and the Faroe Islands) is the correct method. Ryan (2005) conducted this integrated analysis and found a dose–response slope much lower than that of Trasande et al. It should also be noted that to conduct this integrated analysis, Ryan reanalyzed the Faroe Islands data, the data set on which Trasande et al. based their dose–response slope. Ryan found a dose–response slope much lower, in absolute value, than that reported by Trasande et al., further supporting our position that Trasande’s dose–response slope is relatively high, in absolute value.

Another important difference between the assumptions used by the U.S. EPA and Trasande et al. involves the calculation of lifetime earnings. The U.S. EPA used an approach similar to one that it has used for other rules, estimating lifetime earnings for the population as a whole. Although the study used by Trasande et al. (Max et al. 2004) did attempt to produce a population-level average by multiplying the mean annual earnings for full-time, year-round workers by the percent of the population whose major activity in the preceding week was working at a job or business, it is unclear why this approach would be superior to simply obtaining the population-level average. On the other hand, U.S. EPA did not appear to include the value of wage supplements or nonmarket household production, which should be considered in lifetime earning calculations.

Both analyses include similar approaches to assess the impact a decrement in IQ has on lifetime earnings. Although Trasande included a sex-specific approach that more closely follows Salkever (1995), the U.S. EPA’s participation-weighted approach produced nearly the same result. On the other hand, the U.S. EPA included the impact that IQ decrements have on the years of schooling. Although the impact of including this factor is probably very small, it is technically appropriate.

The last two sets of assumptions concern the percentage of fish consumption affected by domestic and global deposition, and the percentage of global and domestic deposition affected by U.S. sources. A spatially explicit model of air quality and deposition is clearly preferable, but this type of modeling is both difficult and expensive. Thus, broad assumptions, such as those in Trasande’s analysis, are sometimes necessary. That said, Trasande’s particular assumption that 60% of U.S. deposition is attributed to U.S. sources seems implausibly high in light of U.S. EPA’s air dispersion modeling results, which suggest a figure of approximately 16%. We also believe that the percentage of fish consumption affected by global and domestic sources is more accurately estimated using consumption data as opposed to landings data, which ignores some very important location issues.

Finally, we end with three important caveats. First, in this analysis we evaluate decrements in IQ associated with prenatal mercury exposure, and monetize these results by evaluating changes in lifetime earnings. In this case, IQ is being used as a surrogate for other subtle neurobehavioral end points. We do not address any other possible health outcomes from mercury exposure (e.g., cardiovascular effects), nor do we address other possible issues associated with IQ decrements, such as increased cases of mental retardation as pointed out by Trasande et al. (2006a, 2006b). Second, Trasande’s analysis includes a threshold for mercury impacts. In other words, prenatal exposure to mercury from mothers who have a blood mercury level < 4.84 μg/L is estimated to have no impact. The U.S. EPA’s upper-bound estimate of $210 million per year assumed no threshold. All prenatal exposure was assumed to have an impact. This is one of the reasons why introducing the U.S. EPA assumptions in Trasande’s model produced a monetized impact for U.S. power plant emissions of $10 million per year, rather than an estimate closer to the $210 million per year. Finally, even though it has been stated a number of times that the results of Trasande’s analysis cannot be considered a benefits estimate of mercury reduction, in fact they are often used this way. One must remember that to estimate benefits, one must include a measure of the ecosystem response time and a discount rate.

## Figures and Tables

**Table 1:** Trasande’s base case linear model of global anthropogenic mercury emissions, using the corrected dose–response slope, with a cord:maternal blood ratio of 1.7.

	Segment of the population (%)			
Characteristic	90–92.1	92.2–94.9	95–99.3	≥ 99.4
Hg concentration range (μg/L)	4.84–5.8	5.8–7.13	7.13–15.0	> 15.0
Maternal Hg concentration (μg/L)	4.84	5.8	7.13	15
No effect concentration (μg/L)	3.41	3.41	3.41	3.41
Change in concentration (μg/L)	2.431	4.063	6.324	19.703
Dose–response slope	0.093	0.093	0.093	0.093
IQ points lost	0.23	0.38	0.59	1.83
Lifetime earnings (US$ 2000)				
Boys	1,032,002	1,032,002	1,032,002	1,032,002
Girls	763,468	763,468	763,468	763,468
Decrease in lifetime earnings for loss of 1 IQ point (%)				
Boys	1.93	1.93	1.93	1.93
Girls	3.23	3.23	3.23	3.23
No. of births				
Boys	45,693	58,155	91,387	12,462
Girls	43,601	55,492	87,201	11,891
EAF (%)	70.00	70.00	70.00	70.00
Economic impact (US$ 2000)				
Boys	140 million	310 million	750 million	320 million
Girls	170 million	360 million	880 million	380 million
Total (US$ 2000)	310 million	670 million	1.6 billion	700 million

**Table 2:** Comparison of the monetized impact of IQ decrements from anthropogenic mercury emissions under assumptions by Trasande et al. and the U.S. EPA.

Monetized impacts	Trasande (original)	Trasande (corrected)	U.S. EPA
Undiscounted effects ($US 2000)			
Monetized impact of anthropogenic emissions	33 billion	3 billion	580 million
Monetized impact of U.S. anthropogenic emissions	12 billion	1 billion	35 million
Monetized impact of U.S. power plant emissions	5 billion	480 million	15 million
Discounted effects ($US 2000)			
Monetized impact of anthropogenic emissions	33 billion	3 billion	370 million
Monetized impact of U.S. anthropogenic emissions	12 billion	1 billion	25 million
Monetized impact of U.S. power plant emissions	5 billion	480 million	10 million
Assumptions			
Linear dose–response slope	0.93	0.093	0.032
Male lifetime earnings ($US 2000)	1,032,002	1,032,002	472,465
Female lifetime earnings ($US 2000)	763,468	763,468	472,465
Male earning loss of 1 IQ point decrement (%)	1.93	1.93	2.38
Female earning loss for 1 IQ point decrement (%)	3.23	3.23	2.38
Fish consumption affected by U.S. deposition (%)	58	58	30
Fish consumption affected by global sources (%)	42	42	70
Domestic deposition from U.S. sources (%)	60	60	16
Global deposition from U.S. sources (%)	2	2	2
U.S. emissions from U.S. power plants (%)	41	41	41
Discount rate (%)	0	0	3
Average no. of years for ecosystem adjustment	0	0	15

**Table 3:** Sensitivity analysis of the impact of U.S. EPA assumptions on the Trasande (corrected) results.

	Impact of U.S. EPA assumptions	
Assumptions	Global estimate	U.S. estimate
Linear dose–response slope (%)	−66	−66
Male and female lifetime earnings (%)	−46	−46
Male and female earning loss for 1 IQ point decrement (%)	−4	−4
Fish consumption affected by U.S. and global deposition (%)	0	−46
Domestic and global deposition from U.S. sources (%)	0	−72
U.S. emissions attributable to U.S. power plants (%)	Unchanged	
Discount rate and average no. of years for ecosystem adjustment (%)	−36	−36
All assumptions (%)
Undiscounted effects	−81	−97
Discounted effects	−88	−98
